# P-1138. Assessing Clinically Relevant Environmental Bacterial and Viral Contamination in Neonatal Care Areas

**DOI:** 10.1093/ofid/ofaf695.1332

**Published:** 2026-01-11

**Authors:** Bobby G Warren, Amanda M Graves, Aaron Barrett, Guerbine Fils-Aime, isadora Mamikunian, Melissa Campbell, Lakshmi Katakam, Deverick J Anderson, Ibukunoluwa Kalu

**Affiliations:** Duke University School of Medicine, Hillsborough, North Carolina; Duke University School of Medicine Duke Center for Antimicrobial Stewardship and Infection Prevention, Durham, NC; Duke Health, Cary, North Carolina; Duke School of Medicine, Durham, North Carolina; Duke University, San Francisco, California; Duke University Medical Center, Durham, North Carolina; Duke university, Durham, North Carolina; Duke Center for Antimicrobial Stewardship and Infection Prevention, Durham, NC; Duke University, San Francisco, California

## Abstract

**Background:**

Despite prolonged hospitalization of premature neonates and numerous high-touch surfaces in Neonatal Intensive Care Units (NICU), the extent of environmental contamination with bacteria and viruses remains poorly characterized.

**Table 1.**
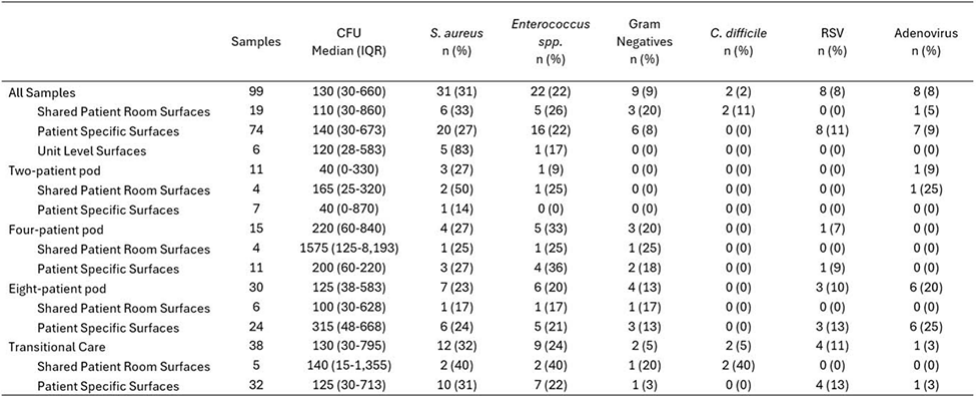
Environmental Samples’ Bioburden and proportion positive for target pathogens by NICU room type and sample surface category NICU, Neonatal Intensive Care Unit; CFU, Colony Forming Units; S. aureus, Staphylococcus aureus; C. difficile, Clostridiodes difficile; RSV, Respiratory Syncytial Virus

**Figure:**
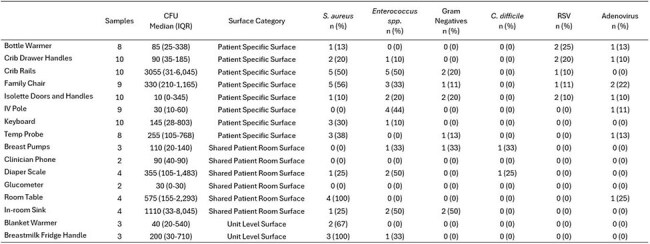
Environmental Sample Bioburden and Target Identification by Neonatal Intensive Care Units’ surfaces

**Methods:**

We completed a prospective microbiological analysis of high-touch surfaces in two NICU settings at a tertiary care center over a 2-week period: shared patient pods housing 2, 4 or 8 patients in a Level IV NICU and a low acuity level 2 NICU that houses up to 15 patients. In each care area, flocked swabs were used to collect samples from a variety of surface categories: patient-specific (PSS), shared within patient rooms (SPR), and shared across the unit (SU). Samples were plated on multiple media for overall and target bacterial species and underwent qPCR for virus detection. Target pathogens included *S aureus*, *Enterococcus spp*., Gram Negative bacteria, *C. difficile*, RSV, and Adenovirus.

**Results:**

A total of 99 samples were obtained; 74 (75%) from PSS, 19 (19%) from SPR, and 6 (6%) from SU. The median overall bioburden for all samples was 130 CFU (IQR: 30-660) and was generally similar between room types and surface categories. In total, 56 (57%) samples were positive for any target pathogen: 41 (55%) from PSS, 10 (53%) from SPR, and 5 (83%) from SU. Targeted bacteria, except *C. difficile*, were recovered similarly amongst room type and surface categories (Table 1). *C. difficile* was only recovered twice but both samples came from SPR; a diaper scale and a breast pump. RSV and Adenovirus were detected in 8 (8%) and 8 (8%) of samples, respectively. Of those, 8 (100%) for RSV and 7 (88%) for Adenovirus were detected from PSS samples (Table 2). Of the 34 patients housed in rooms where PSS and SPR samples were taken, none had a current infection or positive microbiological culture within 30 days prior to sampling.

**Conclusion:**

Despite low overall bioburden, over half of samples (57%) were positive for at least one target pathogen, indicating that microbial contamination persists even in seemingly clean NICU environments. Bacterial detection rates were similar across room type and surface category; however, RSV and adenovirus were identified almost exclusively on PSS. These findings highlight the need for larger studies focused on environmental surveillance in NICUs.

**Disclosures:**

All Authors: No reported disclosures

